# Oseltamivir Expands Quasispecies of Influenza Virus through Cell-to-cell Transmission

**DOI:** 10.1038/srep09163

**Published:** 2015-03-16

**Authors:** Kotaro Mori, Kensaku Murano, Ryosuke L. Ohniwa, Atsushi Kawaguchi, Kyosuke Nagata

**Affiliations:** 1 Department of Infection Biology, Faculty of Medicine and Graduate School of Comprehensive Human Sciences, University of Tsukuba, Tsukuba, Japan; 2 Division of Biomedical Science, Faculty of Medicine and Graduate School of Comprehensive Human Sciences, University of Tsukuba, Tsukuba, Japan; 3 Faculty of Medicine, University of Tsukuba, Tsukuba, Japan

## Abstract

The population of influenza virus consists of a huge variety of variants, called quasispecies, due to error-prone replication. Previously, we reported that progeny virions of influenza virus become infected to adjacent cells via cell-to-cell transmission pathway in the presence of oseltamivir. During cell-to-cell transmission, viruses become infected to adjacent cells at high multiplicity since progeny virions are enriched on plasma membrane between infected cells and their adjacent cells. Co-infection with viral variants may rescue recessive mutations with each other. Thus, it is assumed that the cell-to-cell transmission causes expansion of virus quasispecies. Here, we have demonstrated that temperature-sensitive mutations remain in progeny viruses even at non-permissive temperature by co-infection in the presence of oseltamivir. This is possibly due to a multiplex infection through the cell-to-cell transmission by the addition of oseltamivir. Further, by the addition of oseltamivir, the number of missense mutation introduced by error-prone replication in segment 8 encoding NS1 was increased in a passage-dependent manner. The number of missense mutation in segment 5 encoding NP was not changed significantly, whereas silent mutation was increased. Taken together, we propose that oseltamivir expands influenza virus quasispecies via cell-to-cell transmission, and may facilitate the viral evolution and adaptation.

RNA viruses exist as genetically diverse populations, termed quasispecies, due to error-prone replication by viral RNA-dependent RNA polymerases^1^,^2^,^3^. Although the high mutation rate carries a substantial risk of production of defective progeny viruses, the expansion of virus quasispecies is a great opportunity for viruses to evolve and escape from antiviral drugs^4^,^5^,^6^,^7^,^8^, neutralizing antibody^9^, and cytotoxic T-cell^10^,^11^. It is also reported that virus quasispecies are associated with pathogenesis such as central nervous system infection of poliovirus^12^,^13^. Mathematical models predicted that virus quasispecies are not just collection of different kind of mutants, but a group of interactive variants, which contribute to the characteristics of the population together^14^.

Influenza virus is one of the most serious zoonotic pathogens causing seasonal epidemics and periodic pandemics among human beings around the world. The genome of influenza virus consists of eight-segmented and single-stranded RNAs. The segmented structure of the virus genome allows genetic reassortment when more than two genetically different viruses co-infect a single cell. The viral envelope consists of a lipid bilayer derived from cellular plasma membrane with two viral spike proteins, hemagglutinin (HA) and neuraminidase (NA). Influenza virus infection is initiated by the attachment of HA on virus particles to terminal sialic acid residues in glycoconjugates^15^. NA possesses the enzymatic activity that cleaves α-ketosidic linkages between terminal sialic acids and adjacent sugar residues^16^. In the absence of the functional sialidase activity, progeny virions are not released from sialic acids on the plasma membrane and found to be aggregated on the cell surface^17^,^18^. Further, it is known that this activity contributes to prevention of successive super-infection of infected cells^19^.

Viruses are released as cell-free virions from an infected cell and become infected to distant cells and tissues. In contrast, the virus transmission mechanism from an infected cell to adjacent cells without viral diffusion into the extracellular environment, termed cell-to-cell transmission, has been reported in several viruses^20^,^21^. In general, influenza virus becomes infected from an infected cell to uninfected cells as cell-free virions. Previously, we reported that cell-to-cell transmission of influenza virus also occurs in the presence of oseltamivir which is a potent inhibitor of sialidase activity of NA^22^,^23^,^24^. Since progeny virions are enriched on the plasma membrane of an infected cell in the presence of oseltamivir, it is assumed that viruses become infected to adjacent cells at high multiplicity of infection via cell-to-cell transmission.

Here, we showed that temperature-sensitive (*ts*) mutations remain in progeny viruses even at non-permissive temperature by co-infection in the presence of oseltamivir. This is possibly due to a multiplex infection through the cell-to-cell transmission by the addition of oseltamivir. Further, Next-Generation sequencing analyses revealed that spontaneous mutations introduced by error-prone replication were accumulated in the virus population by oseltamivir treatment. In conclusion, we propose that oseltamivir expands the virus quasispecies via cell-to-cell transmission, and cell-to-cell transmission may contribute to the evolution of influenza virus.

## Results

### Survival of defective variants by oseltamivir treatment

Virus quasispecies are diversified by the error-prone virus genome replication. Defective mutations are generated, but defective viruses can survive in the virus quasispecies population through incidental infection of wild-type virus or viruses containing different type of mutation(s) as helper virus. Thus, it is suggested that high multiplicity of infection is important for the expansion of virus quasispecies. Recently, we found that progeny influenza virions become infected to the adjacent cells via cell-to-cell transmission in the presence of oseltamivir possibly with high multiplicity of infection^23^. Thus, we hypothesized that oseltamivir maintains defective variants in virus quasispecies population by co-infection.

**Figure 1:**
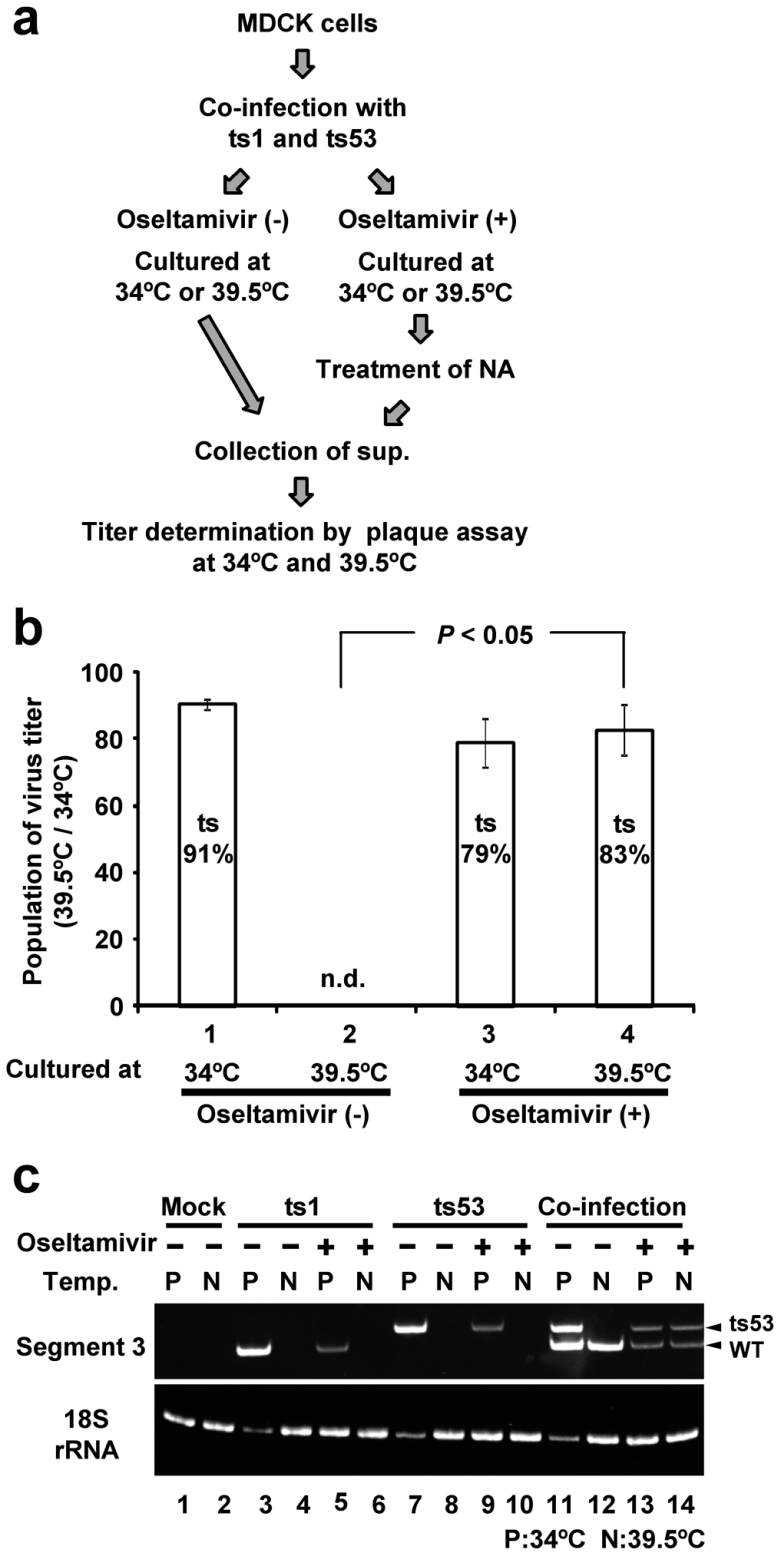
Survival of temperature sensitive mutants in the presence of oseltamivir. (a) Experimental scheme. MDCK cells were co-infected with *ts*1 and *ts*53, and then cultured at 34°C or 39.5°C in the absence or presence of 50 μg/ml of oseltamivir. Viruses grown in the presence of oseltamivir were harvested from the cell surface by treatment with bacterial NA. (b) Phenotyping by plaque assay. The culture supernatant was collected at 60 hpi, and then its virus titer was determined by plaque assays under both 34°C and 39.5°C. Population of *ts* mutant was calculated by the virus titer (T) represented in Table 1 using following formula, {1 − T (39.5°C)/T (34°C) c*100. The population of *ts* mutant treated with oseltamivir (lane 4) was compared with that of untreated one (lane 2) (*P* < 0.05). Error bars indicate s.d. from 3 independent experiments. (c) Genotyping by *Stu* I digestion. Total RNA was extracted at 40 hpi, and RT-PCR was performed using primer set for segment 3. Subsequently, the amplified DNA products were digested with *Stu* I and separated on 8% native-PAGE. Large and small fragments derived from *ts*53 and *ts*1, the latter of which has the same genome sequence of segment 3 as that of wild-type virus, were 220 and 199 base pairs, respectively. P and N indicate permissive temperature (34°C) and non-permissive temperature (39.5°C), respectively. The image of full length gel is represented in Supplementary Figure 2.

To examine the hypothesis, we carried out co-infection experiments using temperature-sensitive (*ts*) mutant viruses, *ts*1 and *ts*53. The viral transcription of *ts*1 is defective at non-permissive temperature due to a mutational change in PB2 polymerase subunit at the amino acid position of 417 from Asp to Asn^25^,^26^. A point mutation causing the change from Leu to Pro at the amino acid position of 226 in *ts*53 PA polymerase subunit results in the defective virus genome replication at non-permissive temperature^27^,^28^,^29^. Since these point mutations are located in different virus genome segments, wild-type virus can be produced by reassortment between *ts*1 and *ts*53 during co-infection. To perform co-infection experiments using *ts*1 and *ts*53 efficiently, 0.1% confluent Madin-Darby canine kidney (MDCK) cells were co-infected with *ts*1 and *ts*53 at each multiplicity of infection (MOI) of 5 (Fig. 1a). In order to facilitate the infection of progeny viruses from co-infected cells to adjacent uninfected cells, fresh MDCK cells were overlaid onto co-infected cells confluently after virus adsorption, followed by incubation at either permissive (34°C) or non-permissive temperature (39.5°C) in the absence or presence of 50 μg/ml of oseltamivir. Oseltamivir at the concentration of 50 μg/ml completely blocks the release of progeny influenza viruses^23^. The culture medium including progeny virions was collected at 60 hours post infection (hpi). The viral titers of wild-type and *ts* mutant viruses were determined by plaque assays at permissive and non-permissive temperatures (Fig. 1a). It is reported that there are several mutations in NA conferring resistance to oseltamivir^30^, but both of *ts* mutant viruses did not have such mutations (Supplementary Fig. 1). The virus titer determined by plaque assays at 34°C indicates those including *ts* mutant viruses and wild-type virus, while the virus titer determined at 39.5°C indicates that for wild-type virus alone. Therefore, the ratio of virus titers between 34°C and 39.5°C means a population rate of *ts* mutant viruses. During infection with single *ts* mutant, cells that were infected with *ts* mutant and incubated at 34°C and 39.5°C did not produce any wild-type virus that could grow at 39.5°C in plaque assay (Table 1). Co-infection with *ts*1 and *ts*53 at permissive temperature without oseltamivir produced 1.2 × 10^5^ plaque forming unit (PFU)/ml of wild-type virus possibly due to segment reassortment. The virus titer determined at 34°C was 1.3 × 10^6^ PFU/ml including both *ts* mutant viruses and wild-type virus. Thus, the population rate of *ts* virus was calculated to be 91% (Table 1 and Fig. 1b). At non-permissive temperature, co-infection with *ts*1 and *ts*53 without oseltamivir produced 1.6 × 10^5^ PFU/ml of wild-type virus. The virus titer determined at 34°C was 1.5 × 10^5^ PFU/ml including *ts* mutant viruses and wild-type virus, suggesting that the production of *ts* mutant viruses hardly occurs in the absence of oseltamivir. On the other hand, co-infection with *ts*1 and *ts*53 in the presence of oseltamivir at non-permissive temperature produced 6.9 × 10^3^ PFU/ml of wild-type virus, while the virus titer determined at 34°C including *ts* mutant viruses and wild-type virus was 4.3 × 10^4^ PFU/ml. Thus, it is calculated that the population rate of *ts* mutant was 83% by co-infection with *ts*1 and *ts*53 with oseltamivir treatment even cultured at non-permissive temperature.

To further confirm this, total RNA was purified from infected cells at 40 hpi, and cDNA fragments amplified from segment 3 viral RNA were digested by *Stu* I as reported previously^23^. *ts*53 virus has a substitution mutation from U to C at the nucleotide position of 701 in segment 3 encoding PA, while the sequence of segment 3 of *ts*1 virus is the same as that of wild-type virus. The digested DNA fragments containing 220 and 199 base pairs were derived from *ts*53 and wild-type viruses, respectively. As expected, the virus genome derived from *ts*53 was clearly found even at non-permissive temperature in the presence of oseltamivir (Fig. 1c), suggesting that defective mutations are maintained in the virus population produced in the presence of oseltamivir.

**Figure 2:**
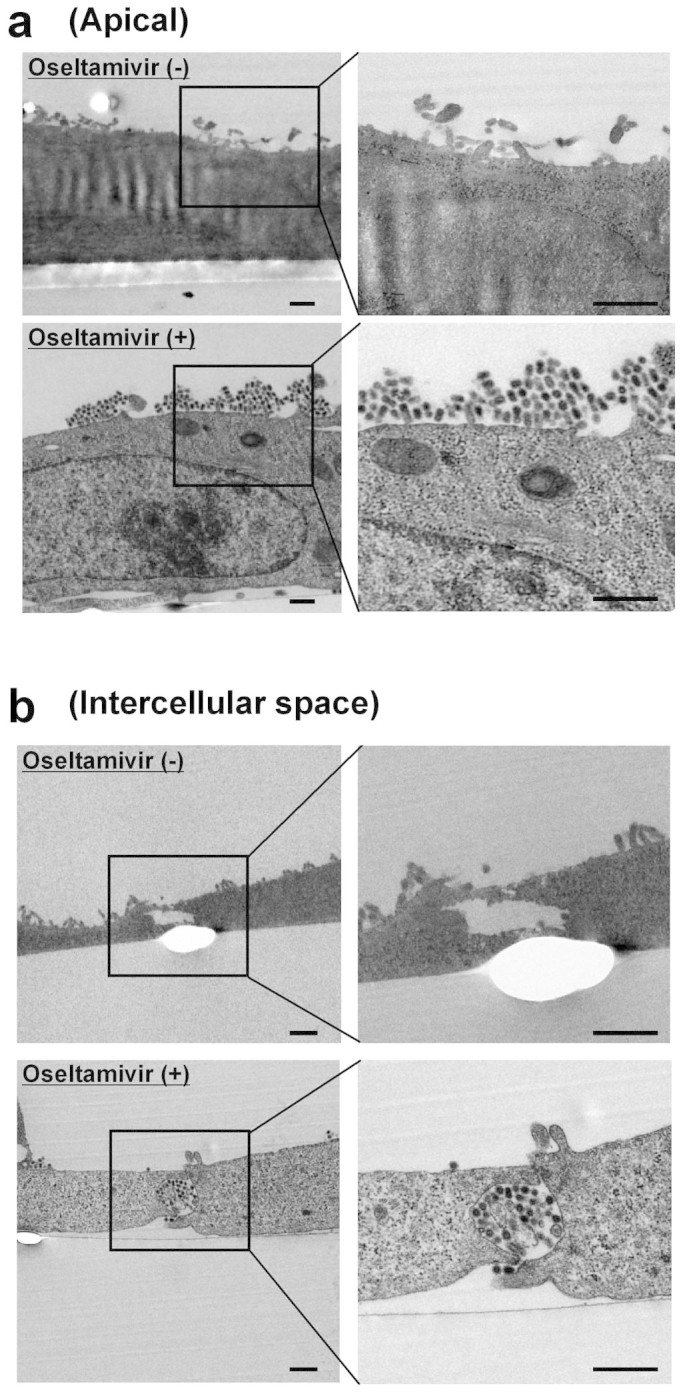
Prominent localization of influenza virus in intercellular space in the presence of oseltamivir. MDCK cells were infected with influenza virus WSN/33 at MOI of 10 and cultured in the absence or presence of 50 μg/ml of oseltamivir phosphate. After incubation at 37°C for 16 hours, transmission electron microscopic analyses were performed. (a) Virions accumulated on apical plasma membrane. (b) Virions accumulated in intercellular space. Enlarged views are shown in borders. Scale bar, 500 nm.

Influenza virus particles are enriched on the cell surface by inhibiting the virus release from the infected cells with oseltamivir^17^,^18^. These accumulated progeny virions may become infected to adjacent cells via cell-to-cell transmission^23^. To support this, we carried out transmission electron microscopic analyses with or without 50 μg/ml oseltamivir. As reported previously^31^, large clusters of progeny virions were attached to the apical plasma membrane by oseltamivir treatment (Fig. 2a). Further, we found that, in the presence of oseltamivir, a significant amount of the progeny virions accumulated in intercellular space (Fig. 2b). Thus, it is possible that accumulated progeny virions present in intercellular space become infected to adjacent cells via cell-to-cell transmission in the presence of oseltamivir. Taken these results together, progeny virions containing defective virus genomes could be rescued by co-infection with helper viruses at the high multiplicity of infection via cell-to-cell transmission.

### Diversity of virus quasispecies in the presence of oseltamivir

**Figure 3:**
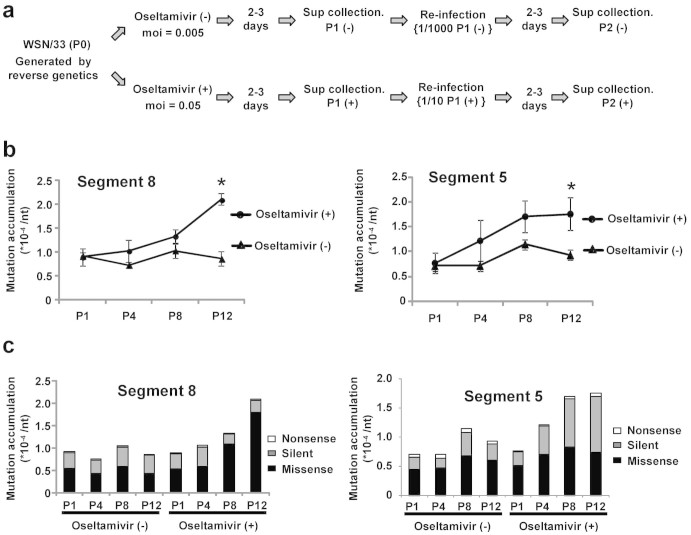
Expansion of virus quasispecies by oseltamivir. (a) Experimental scheme. MDCK cells were infected with wild-type virus at MOI of 0.005 or 0.05 and cultured in the absence or presence of 50 μg/ml of oseltamivir. When CPE was observed, the culture supernatant was collected and designated P1. Viruses grown in the presence of oseltamivir were harvested from the cell surface using bacterial NA. Supernatant P1 obtained in the absence or presence of oseltamivir were diluted 10^3^ times and 10 times, respectively and employed for infection in the second round of passage. After infection, cells were cultured in the absence or presence of 50 μg/ml oseltamivir again. This procedure was repeated 12 times. (b and c) Ratio of mutation accumulated in virus genome. Viral RNA was collected from supernatant P1, P4, P8, and P12, and the cDNA of segment 8 and 5 between the nucleotide positions of 41 to 386 and 217 to 552, respectively were amplified by RT-PCR. After gel purification, cDNAs were subjected to Next-Generation sequencing, and the ratio of mutation accumulated in virus segment was calculated (b). The ratio of the mutation accumulation in P12 virus treated with oseltamivir was compared with that of P12 virus which was not treated. *, *P* < 0.05. Error bars indicate s.d. from 3 independent experiments. The number of nonsense, silent, and missense mutation in NS1 encoded by segment 8 and NP encoded by segment 5 at the amino acid positions of 13 to 113 and 65 to 162 was counted, respectively (c).

The fidelity of the influenza viral RNA-dependent RNA polymerase is quite low, and therefore generates virus quasispecies^32^,^33^,^34^. In the presence of oseltamivir, *ts* mutant viruses were maintained in the virus quasispecies population via cell-to-cell transmission (Fig. 1). Based on this, we hypothesized that spontaneous mutations introduced by the error-prone replication are accumulated in virus quasispecies population generated via cell-to-cell transmission in the presence of oseltamivir. To address this, serial virus passages were carried out in the presence or absence of oseltamivir, and mutations were detected by Next-Generation sequencing (NGS). We generated a recombinant WSN/33 virus by a plasmid-driven reverse genetics method^35^ to prepare a monoclonal parent strain as passage 0 (P0) virus. MDCK cells infected with P0 virus were cultured in the absence or presence of 50 μg/ml of oseltamivir. When cytopathic effects (CPE) were observed, the culture supernatant containing P1 virus was collected and subjected to the second round infection. These processes were serially repeated 12 times (Fig. 3a). The mutation related with the resistance to oseltamivir was not found in P12 virus which was treated with oseltamivir (Supplementary Fig. 1). After reverse transcription of viral RNA purified from P1, P4, P8, and P12 viruses, cDNAs of segment 8 and segment 5 between the nucleotide positions of 41 to 386 and 217 to 552, respectively were amplified by PCR. To evaluate the intrinsic error rate associated with NGS, segment 8 cDNA was also amplified by PCR using plasmid as a template and subjected to NGS. The error rate of NGS was 3.4 × 10^−5^/nt. Thus, we subtracted the error rate of NGS from the mutation rate obtained from each sample. Without oseltamivir treatment, the number of mutation in virus quasispecies population was not changed along with serial passages. In contrast, the mutation rates of both segment 8 and segment 5 were clearly increased in a passage-dependent manner by the addition of oseltamivir (Fig. 3b). These results indicated that the spontaneous mutations introduced by the error-prone replication are accumulated in virus quasispecies population in the presence of oseltamivir. Nucleotide substitution results in missense, silent, or nonsense mutation. Without oseltamivir treatment, the number of missense, silent, and nonsense mutations was not changed significantly. On the other hand, the number of missense mutation of P8 and P12 virus in segment 8 was clearly increased in the presence of oseltamivir (Fig. 3c). In the case of segment 5, the number of silent mutation was significantly increased, but the number of missense mutation was not increased dramatically. Collectively, it is considered that the mutations introduced by the error-prone replication are propagated in virus quasispecies in the presence of oseltamivir.

## Discussion

Here, we demonstrated that oseltamivir expands the influenza virus quasispecies through the cell-to-cell transmission. By the addition of oseltamivir, the number of missense mutation in segment 8 was increased in a passage-dependent manner. The number of missense mutation in segment 5 was not changed significantly, whereas silent mutation in segment 5 was increased (Fig. 3c). It is well known that NS1 encoded by segment 8 is tolerant of amino acid changes, while NP encoded by segment 5 is strongly restricted^36^. NS1 inhibits the cellular gene expression and prevents the activation of interferon system^37^. Further, NS1 selectively enhances the translation of viral mRNA and may regulate the synthesis of viral RNA indirectly^37^. Although these functions of NS1 contribute to effective virus production, NS1 is not necessary for virus production^38^, suggesting that the competition between functional and dysfunctional NS1, if any, is not completely a serious problem. However, NP plays a key role for the virus genome replication and necessary for infectious virus production^39^. It is possible that the competition between functional and defective NP possibly as dominant negative mutant and/or decoy could give negative effect on the virus replication. Thus, missense mutations might not be accumulated in segment 5.

In the presence of oseltamivir, progeny influenza virus virions were found not only on apical plasma membrane but also in intercellular space (Fig. 2). It is generally thought that the budding of influenza virus as a cell-free virion occurs only from the apical surface^40^, since HA and NA glycoproteins are intrinsically transported to the apical plasma membrane^41^,^42^. Thus, it is assumed that the virions accumulated on apical plasma membrane might be translocated into intercellular space by trafficking of apical recycling endosomes to the lateral plasma membrane^43^. It is possible that the intercellular space is a tight structure so as to be a barrier to fluid between apical aqueous environment and intercellular space. Thus, it is speculated that virions accumulated in intercellular space become infected to adjacent cells due to decrease in concentration of oseltamivir in intercellular space.

Error-prone replication anticipates the existence of an error threshold for the maintenance of genetic information. Increase of the average error frequency above a critical threshold during virus genome replication could cause the loss of genetic information resulting in the extinction of virus population, called error catastrophe^44^. The existence of a threshold for the mutation rate of virus replication has been reported. Above the 6.4 times increase in mutation rate of foot-and-mouth disease virus leads to viral extinction^45^. Four times increase in polio virus strongly decreases viral infectivity^46^. We demonstrated that the number of mutation in quasispecies of influenza virus population in the presence of oseltamivir increased gradually and reached around twice at P12 virus compared with that at P1. Taken these previous reports and our study together, it is likely that the cell-to-cell transmission in the presence of oseltamivir provides influenza virus with advantage in evolution such as a chance to generate anti-oseltamivir viruses.

It has been reported that the cell-to-cell transmission provides viruses with advantages. Vaccinia virus induces a blocking mechanism of super-infection and thereby become infected to adjacent uninfected cells rapidly^47^. Viral spread via tight cell-cell contacts allows many viruses to escape from neutralizing antibodies thus leading to immune evasion^48^,^49^,^50^. TRIM5α, that is viral restriction factor and effectively inhibits cell-free retroviruses, are less effective to cell-to-cell transmission^51^. Our findings propose a new idea that the cell-to-cell transmission results in expansion of virus quasispecies and may contribute to evolution and adaptation to new environments.

## Methods

### Cells and viruses

MDCK cells were maintained in minimal essential medium (MEM) (Sigma) containing 10% fetal bovine serum. Influenza virus A/WSN/33 and temperature sensitive mutants (*ts*1 and *ts*53)^26^,^27^ were used after single-plaque isolation. MDCK cells were infected with influenza virus A/WSN/33 or *ts* mutants at MOI of 0.01 PFU/cell, and incubated at 37°C and 34°C, respectively. After incubation for 48 h, the culture fluid was harvested and centrifuged at 1,700 × *g* for 10 min. The virus suspension was stored at −80°C until use.

### Co-infection

To examine the transmission of influenza virus from individual co-infected cells to adjacent cells, we carried out co-infection of *ts*1 and *ts*53. *ts*1 and *ts*53 are defective at non-permissive temperature due to a mutation in segment 1 and segment 3, respectively. The segmented genome of influenza virus provides the opportunity to produce reassortant viruses during co-infection in a cell. Since *ts* mutations of *ts*1 and *ts*53 are located in different virus genome segments, wild-type virus was produced by reassortment between *ts*1 and *ts*53. For efficient co-infection of *ts*1 and *ts*53, MDCK cells at 0.1% confluency were co-infected with *ts*1 and *ts*53 at each MOI of 5. After virus adsorption at 37°C for 1 hour, cells were washed with serum-free MEM, and fresh MDCK cells were overlaid onto co-infected cells confluently to examine the transmission of progeny viruses from co-infected cells to adjacent cells. After incubation for 6 hours at either permissive (34°C) or non-permissive temperature (39.5°C) in the absence or presence of 50 μg/ml of oseltamivir phosphate, cells were washed with serum-free MEM. Then, maintenance medium (MEM containing vitamins and 0.1% BSA) was added with or without 50 μg/ml of oseltamivir phosphate and cultured at either 34°C or 39.5°C. At 60 hpi, the culture supernatant was collected, and then its virus titer was determined by plaque assays under both 34°C and 39.5°C. Viruses grown in the presence of oseltamivir were harvested from the cell surface by treatment with 30 mU/ml of bacterial NA derived from *Clostridiun perfringens* (SIGMA).

### RT-PCR

*ts*53 virus has a substitution mutation from U to C at the nucleotide position of 701 in segment 3 encoding PA, while the sequence of segment 3 of *ts*1 virus is the same as that of wild-type virus. To discriminate the genome of *ts*1 (wild-type) and that of *ts*53, total RNA was reverse-transcribed by SuperScript III (Invitrogen) with PA-895-rev (5′-TTAATTTTAAGGCATCCATCAGCAGG-3′), which is complementary to positive-sense RNA of the segment 3. The cDNA was amplified by PCR using primers, PA-895-rev and PA-695-cut (5′-TCTCCCGCCAAACTTCTCAGGCC-3′) partially corresponding to positive-sense RNA of segment 3 between nucleotide sequence positions 678 to 700 except for nucleotide positions 696 and 697. Since segment 3 of *ts*53 has a substitution mutation from U to C at the nucleotide position of 701, PCR products derived from wild-type could be digested by *Stu* I but not that from *ts*53. After PCR reactions, PCR products were digested with *Stu* I and separated on native-PAGE. Large and small fragments derived from *ts*53 and wild-type viruses were 220 and 199 base pairs, respectively^23^. DNA was stained with GelRed (BIOTIUM) and visualized by UV illumination.

### Transmission electron microscope (TEM)

MDCK cells were infected with virus at MOI of 10, and cultured in the absence or presence of 50 μg/ml of oseltamivir phosphate. After incubation at 37°C for 16 h, cells were subsequently fixed with 2.5% of glutaraldehyde. After further fixation with 1% OsO_4_ for 1 h, sequential dehydrations with ethanol in a stepwise manner were carried out followed by propylene oxide treatment, and embedded in Poly/Bed 812 (Polysciences). Ultrathin sections were examined with a JEM-1300 (JEOL) operated at 80 kV.

### Next-generation sequencing (NGS)

Amplicon generation and NGS were carried out as previously reported^52^. Briefly, viral RNA was extracted using Mag/Extractor-RNA- (TOYOBO). Total RNA was reverse-transcribed by reverse transcriptase (TOYOBO) with oligonucleotide primers (5′-AGCAAAAGCAGGGTAGATAA-3′) and (5′-AGCAAAAGCAGGGTGACAAA-3′), which are complementary to nucleotide sequences between position 1 to 20 negative-sense RNAs of segment 5 and 8, respectively. The cDNA was amplified by PCR using fusion primers including multiplex identifier tag (MID) and adaptor for 454 sequencing, (5′-CGTATCGCCTCCCTCGCGCCATCAGTACTGAGCTACAGAACAGCTTAACAATAGA-3′) and (5′-CTATGCGCCTTGCCAGCCCGCTCAGTACTGAGCTAACCCTGCATCAGTGAGCACA-3′) corresponding to segment 5 between nucleotide sequence positions 217 to 236 and 533 to 552, respectively. To amplify segment 8 cDNA, primers, (5′-CGTATCGCCTCCCTCGCGCCATCAGAGCACTGTAGTGTGTCAAGCTTTCAGGTAG-3′) and (5′-CTATGCGCCTTGCCAGCCCGCTCAGAGCACTGTAGGTCCATTCTGATACAAAGAGG-3′) corresponding to segment 8 between nucleotide sequence positions 41 to 60 and 366 to 386, respectively were used. PCR products were purified by agarose gel using Fast Gene Gel Extraction Kit (NIPPON Genetics). Amplicons were subjected to ultra-deep pyrosequencing using GsJunior (454 Life Sciences, Roche).

### Data analysis

The output from the GSJunior includes sequence results (FASTA) and quality score for every sequence position in a read. It is known that average quality score of a read is inversely proportional to the number of errors in that read^53^. To suppress the error associated with NGS, we eliminated the reads including quality score below 27 at one sequence position. The reads which were passed through quality filter were aligned by ClustalW as previously reported^54^, and the number of mutation in each sequence position was counted.

## Supplementary Material

Supplementary Information Supplementary Information

## Figures and Tables

**Table 1:** Virus titer cultured under the permissive or non-permissive temperature

Infected virus	Cultured at	Oseltamivir	Virus titer (PFU/ml)	
			34°C	39.5°C
*ts*1	34°C	−	1.4 × 10^6^ ± 9.5 × 10^5^	n.d.
*ts*1	34°C	+	2.9 × 10^4^ ± 6.5 × 10^3^	n.d.
*ts*1	39.5°C	−	n.d.	n.d.
*ts*53	34°C	−	2.2 × 10^6^ ± 8.1 × 10^5^	n.d.
*ts*53	34°C	+	4.6 × 10^4^ ± 2.2 × 10^4^	n.d.
*ts*53	39.5°C	−	n.d.	n.d.
*ts*1 + *ts*53	34°C	−	1.3 × 10^6^ ± 2.8 × 10^5^	1.2 × 10^5^ ± 2.1 × 10^4^
*ts*1 + *ts*53	39.5°C	−	a1.5 × 10^5^ ± 4.3 × 10^4^	a1.6 × 10^5^ ± 6.9 × 10^4^
*ts*1 + *ts*53	34°C	+	2.2 × 10^4^ ± 4.0 × 10^3^	4.6 × 10^3^ ± 1.9 × 10^3^
*ts*1 + *ts*53	39.5°C	+	b4.3 × 10^4^ ± 2.2 × 10^4^	b6.9 × 10^3^ ± 2.7 × 10^3^

n.d.: not detected. ±: s.d. from 3 independent experiments.

^a^*P* > 0.5, compared with the titers between 34°C and 39.5°C.

^b^*P* < 0.05, compared with the titers between 34°C and 39. 5°C.
