# Supplementary material for: Oseltamivir Expands Quasispecies of Influenza Virus through Cell-to-cell Transmission
Source: Sci Rep. 2015 Mar 16;5:9163. doi: 10.1038/srep09163 (PMC4649863; doi:10.1038/srep09163)
Supplement: Supplementary Information [file srep09163-s1.pdf]

Oseltamivir Expands Quasispecies of Influenza Virus through Cell-to-cell Transmission

Kotaro Mori[1], Kensaku Murano[1], Ryosuke L Ohniwa[2],
Atsushi Kawaguchi[1], and Kyosuke Nagata[3]*

[1]Department of Infection Biology, Faculty of Medicine and Graduate School of Comprehensive Human Sciences, University of Tsukuba, Tsukuba, Japan

[2]Division of Biomedical Science, Faculty of Medicine and Graduate School of Comprehensive Human Sciences, University of Tsukuba, Tsukuba, Japan

[3]Faculty of Medicine, University of Tsukuba, Tsukuba, Japan

Corresponding author: Kyosuke Nagata

Faculty of Medicine, University of Tsukuba, 1-1-1 Tennodai,
Tsukuba 305-8575, Japan.

Phone; (Japan +81) 29-853-3233

Fax; (Japan +81) 29-853-3233

Email; knagata@md.tsukuba.ac.jp

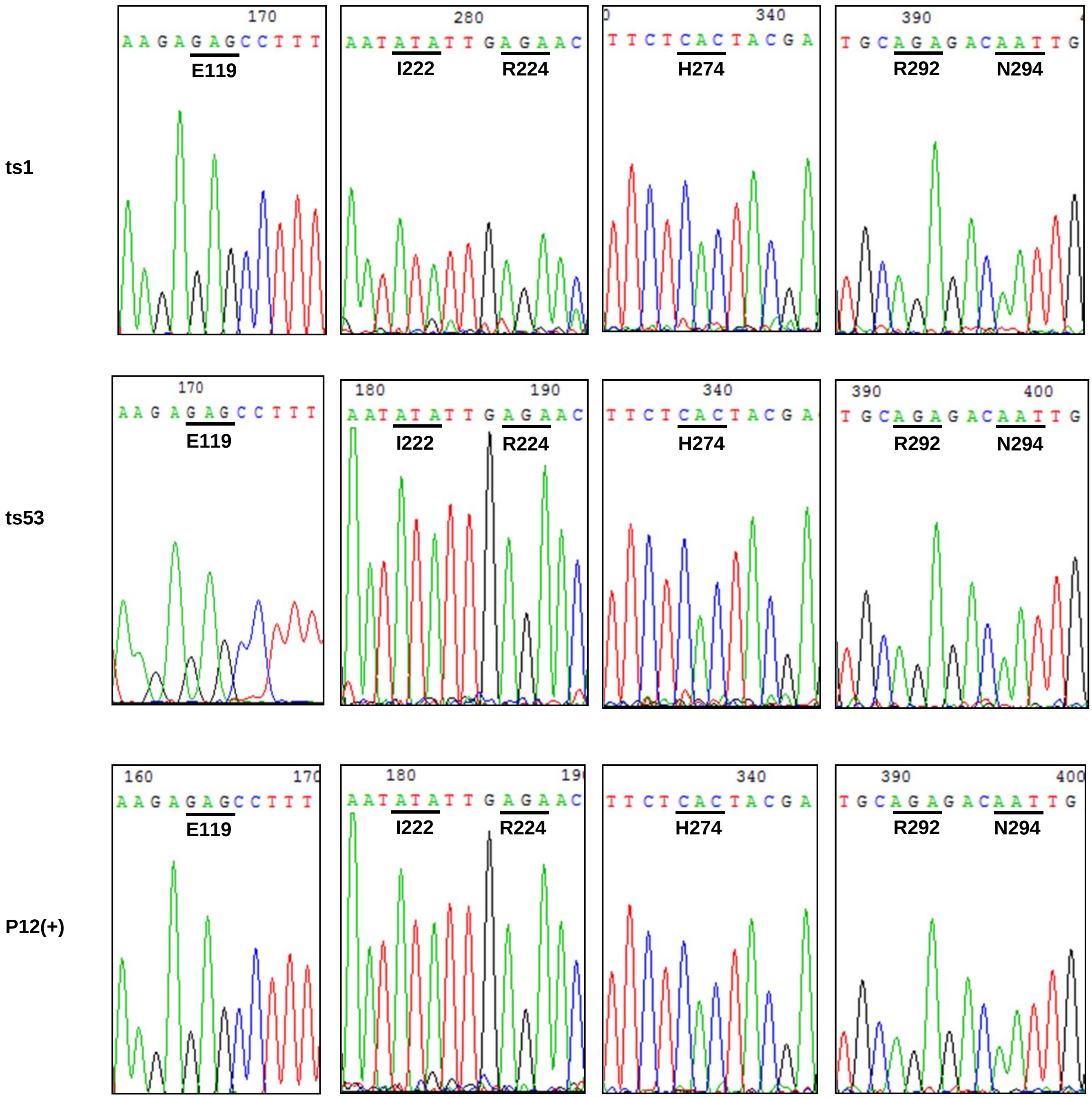

Supplementary Figure 1. **Sequence data related with amino acid involved in oseltamivir-resistant.**

total RNA was reverse-transcribed by reverse transcriptase (TOYOBO) with the primer (5’-TAACCATTGGGTCAATCTGTAT-3’), which is complementary to negative-sense RNA of the segment 6. The cDNA was amplified by PCR using primers, (5’-TAACCATTGGGTCAATCTGTAT-3’), and (5’- GGATCCCAAATCATCTCAAACC-3’) corresponding to segment 6 between nucleotide sequence positions 42 to 63 and 1084 to 1105, respectively. Sequencing was carried out using 3130 Genetic Analyzer (Applied Biosystems)

Segment 3

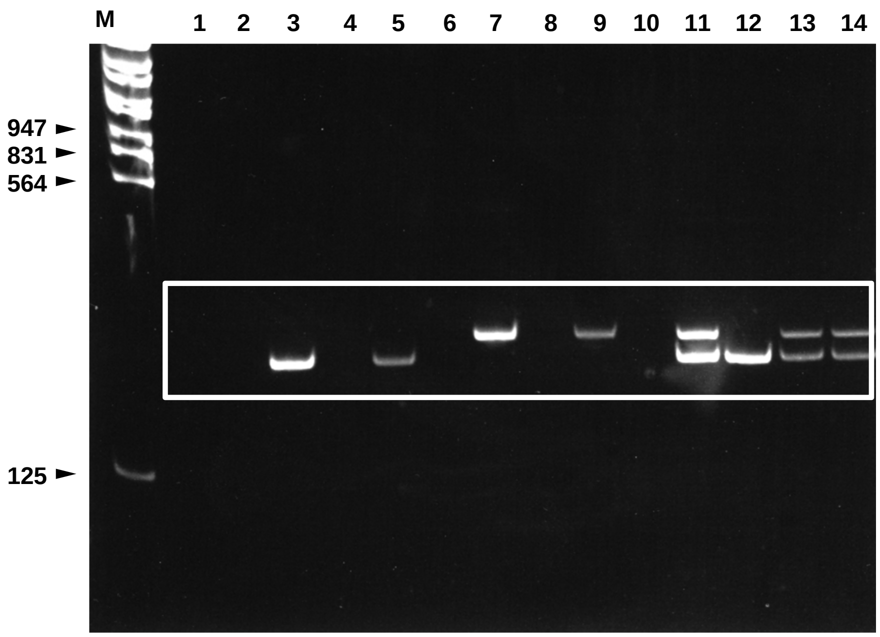

18S rRNA

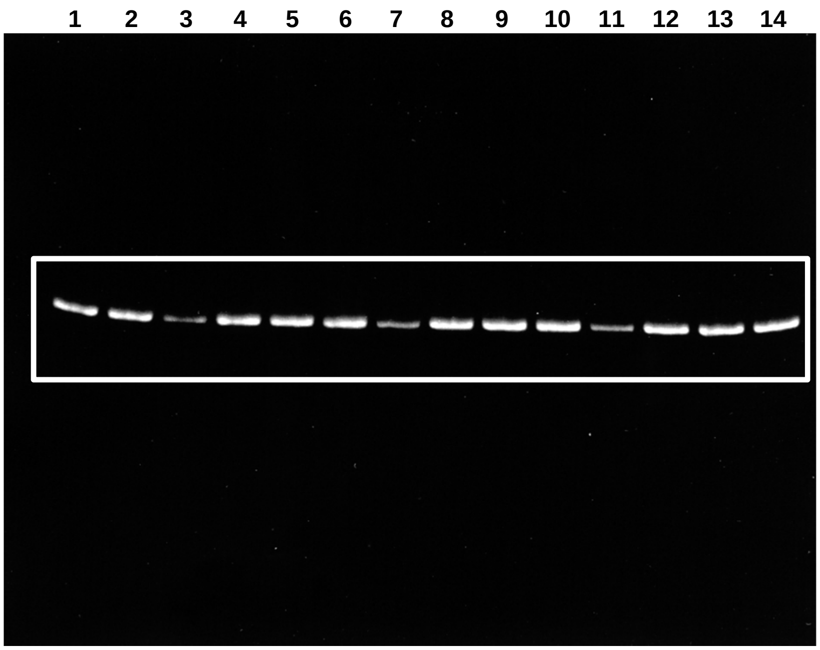

Supplementary Figure 2. Original image of full length gel.

White box indicates cropping line, and represented in Figure 1c. Phage lambda DNA digested with *EcoR* I and *Hind* III was used as marker (M).

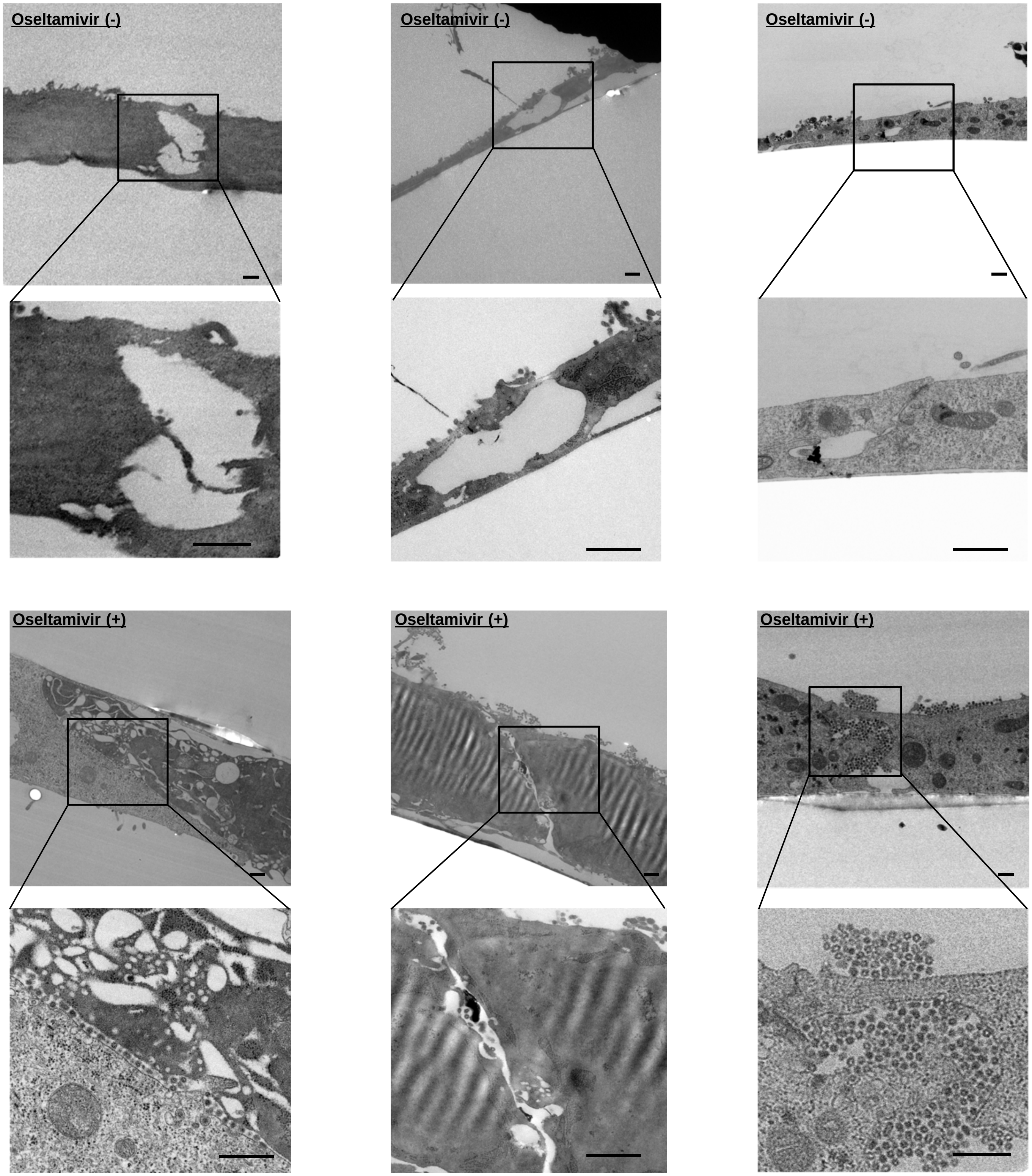

Supplementary Figure 3. Extra photos related with Figure 2b.

Photos showing not only typical but also atypical morphology of the cells untreated or treated with oseltamivir are shown. Enlarged views are shown in borders. Scale bar, 500 nm.
